# Inhibitory effect of rutin and curcumin on experimentally-induced calcium oxalate urolithiasis in rats

**DOI:** 10.4103/0974-8490.75462

**Published:** 2010

**Authors:** Jaydip Ghodasara, Anil Pawar, Chinmay Deshmukh, Bhanudas Kuchekar

**Affiliations:** *Department of Pharmacology, MAEER’s Maharashtra Institute of Pharmacy, Kothrud, Pune - 411 038, Maharashtra, India*

**Keywords:** Calcium oxalate, curcumin, ethylene glycol, rutin, urolithiasis

## Abstract

**Background:**

Renal epithelial cell injury by reactive oxygen species is pre-requisite step in the pathogenesis of urolithiasis. Rutin and curcumin are polyphenolic compounds known to have antioxidant and anti-inflammatory activities, but their effect on urolithiasis is yet to be elucidated. In the present study, we have investigated the inhibitory effect of rutin and curcumin on calcium oxalate urolithiasis in Wistar albino rats.

**Methods:**

Calcium oxalate urolithiasis was induced experimentally by administration of 0.75% v/v ethylene glycol with 1% w/v ammonium chloride in drinking water for three days followed by only 0.75% v/v ethylene glycol for 25 days. Rutin (20 mg/kg body weight) and curcumin (60 mg/kg body weight) were given once daily for 28 days by oral route. After treatment period, calcium and oxalate levels in urine and kidney tissue homogenate were measured. Kidney was also used for histopathological examination.

**Results:**

Stone-induction with ethylene glycol and ammonium chloride resulted in elevated levels of calcium and oxalate in the urine and kidney sample, whereas supplementation of rutin and curcumin restored it near to normal. Histopathological study revealed minimum tissue damage and less number of calcium oxalate deposits in kidney of animal treated with rutin and curcumin as compared to calculi-induced animal.

**Conclusion:**

The data suggest that the rutin and curcumin inhibits calcium oxalate urolithiasis. This effect is mediated possibly through a lowering of urinary concentration of stone forming constituents, anti-inflammatory and antioxidant effects.

## INTRODUCTION

Urinary calculi are the third prevalent disorder in the urinary system.[1] Most calculi in the urinary system arise from a common component of urine, e.g. calcium oxalate representing up to 80% of analyzed stones.[2] Hyperoxaluria is a major risk factor in calcium oxalate stone disease. In rats, hyperoxaluria can easily be induced by various diets. Various approaches have been employed in the previous research to induce urolithiasis including several crystal-inducing drugs, such as ethylene glycol, oxalate, gentamicin sulfate, etc. However, most of these models were associated with nephrotoxicity.[3]

Acute and chronic production of calcium oxalate and crystal deposition induces lipid peroxidation. The generation of lipid peroxidation due to reactive oxygen species causes renal epithelial cell injury which promotes calcium oxalate stone formation by providing cellular debris for crystal nucleation and aggregation, and augments crystal attachment to other tubular cells.[4] Antioxidant therapy with vitamin E, glutathione monoester, methionine, lipoic acid, or fish oil prevented calcium oxalate nucleation and retention in the renal tubules by preventing oxalate-mediated peroxidative injury.[5] The inhibitory effect of some herbals on calcium oxalate urolithiasis is claimed to be due to their antioxidant effects.[6,7] These studies suggest a role of reactive oxygen species in the calcium oxalate stone formation.

Rutin and curcumin have been used for centuries in indigenous medicine for the treatment of a variety of anti-inflammatory conditions and other diseases. They exhibit remarkable anti-inflammatory and antioxidant effects[8,9] and may prevent calcium oxalate associated free radical injury and urolithiasis. Therefore, the present study was undertaken to investigate the inhibitory effect of rutin and curcumin on calcium oxalate urolithiasis in rats.

## MATERIALS AND METHODS

*Animals*: Male Wistar albino rats weighing between 150-200 g each were used for this experiment. They were procured from Agharkar Research Institute, Pune, India. They were housed in polypropylene cages and maintained at 27 ± 2°c, relative humidity 65 ± 10% under 12 h light/dark cycles. The animals were given standard diet supplied by Pranav Agro Industries Ltd, Sangli, India. The study protocol was approved by the Institutional Animal Ethics Committee (Ref. No.: MIP/IAEC/09-10/M1/App-7/003) constituted in accordance with the rules and guidelines of the CPCSEA (Committee for the purpose of Control and Supervision of Experiments on Animals), India.

*Chemicals and apparatus*: Ethylene glycol (B. No.: 75316806-2) was purchased from Qualigens Fine Chemicals, Navi Mumbai, India. Rutin (B. No.: 0000062644) and Curcumin (B. No.: 0000066276) were purchased from Himedia Laboratories Pvt. Ltd., Mumbai, India. All other chemicals and reagents used were of analytical grade and procured from approved vendors. Apparatus such as the metabolic cages (Tecniplast, Italy), cold centrifuge (Remi Instruments, C-308L), and UV-spectrophotometer (CARY 100 Scan, EL08053091) were used in the study.

### Experimental design

Ethylene glycol and ammonium chloride induced hyperoxaluria model was used to induce urolithiasis.[10] Rutin at the dose of 20 mg/kg and curcumin at 60 mg/kg were selected to evaluate their effect on calcium oxalate urolithiasis.[8,11] Twenty-four animals were randomly divided into four groups as Group I, II, III and IV containing six animals each. Group I served as a vehicle-treated control and was maintained on regular rat food and drinking water *ad libitum* and received 0.5% w/v carboxy methyl cellulose solution (5 ml/kg p.o.). All the remaining groups received calculi-inducing treatment for 28 days, comprised of 0.75% v/v ethylene glycol with 1% w/v ammonium chloride in drinking water *ad libitum* for three days to accelerate lithiasis followed by only 0.75% v/v ethylene glycol for 25 days. Group II received 0.5% w/v carboxy methyl cellulose solution (5 ml/kg p.o.). Group III and IV received rutin (20 mg/kg body weight) and curcumin (60 mg/kg body weight) respectively from first day to 28^th^ day of calculi induction. Rutin and curcumin were suspended in water using 0.5% w/v carboxy methyl cellulose and were given once daily by oral route (5 ml/kg body weight).

### Collection and analysis of urine

On the 28^th^ day of calculi induction treatment, all animals were kept in individual metabolic cages and urine samples of 24 h were collected. The collected urine samples were measured for volume, calcium and oxalate contents. The calcium diagnostic kit (Beacon Diagnostics Pvt. Ltd., India. B. No.: LP-996) was used to estimate urinary calcium level, whereas oxalate level was measured according to the previously described method.[12]

### Serum analysis

The blood was collected from the retro-orbital sinus under anesthetic condition and serum was separated by centrifugation at 10,000×g for 10 min and analyzed for creatinine. Serum creatinine levels were estimated by spectrophotometrically using the kit from Span Diagnostics Ltd., India (B. No.: 4000003038).

### Kidney histopathology and homogenate analysis

The abdomen was cut open to remove both kidneys from each animal. Isolated kidneys were cleaned off extraneous tissue, rinsed in ice-cold physiological saline and used for histopathology and homogenate analysis.

The left kidney was finely minced and 20% homogenate was prepared in Tris-Hcl buffer (0.02 mol/l, pH 7.4). Total kidney homogenate was used for assaying tissue calcium, oxalate and lipid peroxidation inhibition activity. The lipid peroxidation inhibition activity in tissue homogenate was estimated according to the previously described method.[13] The calcium kit (Beacon Diagnostics Pvt. Ltd., India. B. No.: LP-996) was used to estimate calcium content in the kidney tissue homogenate. Oxalate level in the kidney tissue homogenate was measured according to the previously described method.[12]

The right kidney was fixed in 10% neutral buffered formalin, processed in a series of graded alcohol and xylene, embedded in paraffin wax, sectioned at 5 μm and stained with Hematoxylin and Eosin for examination under polarized light. The kidney section was also stained by Pizzolato’s method; which selectively stains calcium oxalate crystals for examination under light microscope.[14] The slides were observed to estimate total number of calcium oxalate deposits[7] and tubulointerstitial damage index.[15]

### Statistical analysis

The results were expressed as mean ± standard error mean (SEM). The statistical significance was assessed using one-way analysis of variance (ANOVA) followed by Dunnett’s comparison test and *P*<0.05 was considered significant.

## RESULTS

The urine volume was increased very significantly (*P*<0.01) by calculi-inducing treatment. A co-treatment with rutin and curcumin prevented this increase in urine volume, although urine volume remained higher than those of vehicle control animals [Table 1].

**Table 1:** Effect of rutin and curcumin on urine, kidney and serum parameters in control and experimental animals

Parameters	Group I	Group II	Group III	Group IV
Urine				
Volume (ml/24h)	3.78 ± 0.15	11.16 ± 0.87^a^**	9.91 ± 0.51^a^**	5.08 ± 0.17^b^**
Calcium (mg/24h)	0.17 ± 0.003	0.86 ± 0.011^a^**	0.35 ± 0.014^a^**^b^**	0.20 ± 0.01^b^**
Oxalate (mg/24h)	4.10 ± 0.13	21.26 ± 1.03^a^**	11.70 ± 0.47^a^**^b^**	7.37 ± 0.18^a^**^b^**
Serum				
Creatinine (mg/dl)	0.14 ± 0.003	0.21 ± 0.002^a^**	0.17 ± 0.003^a^**^b^**	0.18 ± 0.002^a^**^b^**
Kidney				
Calcium (mg/g)	0.15 ± 0.003	0.27 ± 0.004^a^**	0.18 ± 0.002^a^**^b^**	0.16 ± 0.003^a^*^b^**
Oxalate (mg/g)	5.52 ± 0.11	11.46 ± 0.15^a^**	7.73 ± 0.18^a^**^b^**	7.76 ± 0.12^a^**^b^**
% lipid peroxidation	14.22 ± 0.38	100.00 ± 0.00^a^**	66.39 ± 0.30^a^**^b^**	21.59 ± 0.31^a^**^b^**
Number of CaOx deposits/100x field	0.00	34.17 ± 1.45^a^**	7.50 ± 0.72^a^**^b^**	9.00 ± 0.82^a^**^b^**

** *P* < 0.01 = very significant

* *P* < 0.05 = significant, Number of animals (N) = 6, Values are expressed as mean ± SEM.

a Comparisons are made with Group I (Vehicle control).

b Comparisons are made with Group II (Lithiatic control)

In the present study, calculi-inducing treatment to male Wistar rats resulted in hyperoxaluria. The calcium and oxalate levels in urine and kidney tissue were significantly (*P*<0.01) increased in calculi-induced animals as compared to vehicle control animals. A co-treatment with rutin and curcumin prevented this increase and restored the calcium and oxalate levels in urine and kidney tissue to near normal Table 1.

Calculi-inducing treatment caused significant increase (*P*<0.01) in lipid peroxidation of kidney tissue, which was prevented in the animals receiving a simultaneous treatment of rutin and curcumin [Table 1].

Kidney of calculi-induced animals showed significant accumulation of calcium oxalate deposits in the kidney tubules. The crystals were large, in groups and seen in tubules of all regions of kidneys: cortex, medulla and papilla. In the kidney of rat treated with rutin and curcumin, visibly small and less abundant crystals were observed compared to those in the kidneys of calculi-induced rats [Table 1].

Calculi-inducing treatment caused impairment of renal functions of the untreated rats as evident from the raised serum creatinine, which was prevented in the animals co-treated with rutin and curcumin [Table 1].

**Figure 1:**
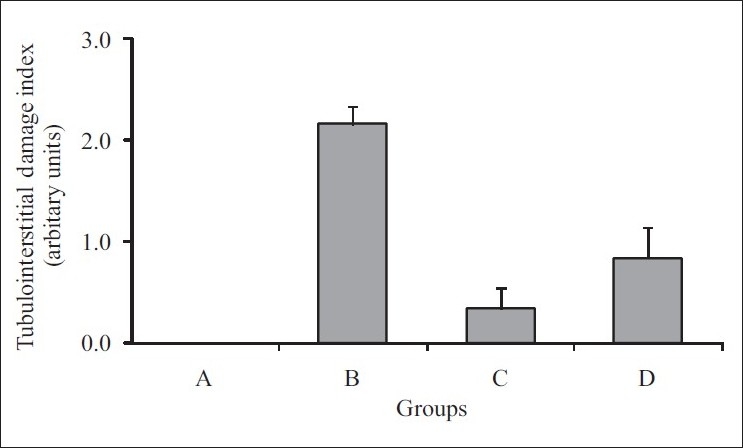
Tubulointerstitial damage index in kidney section of control and treatment groups. A: Vehicle control, B: Lithiatic control, C and D: Rutin (20 mg/kg) and curcumin (60 mg/kg)-treated groups. Values are expressed as mean ± SEM; n=6. Animals treated with rutin and curcumin showed minimal kidney damage compared to the kidneys of lithiatic animals (AT COLUMN WIDTH)

**Figure 2:**
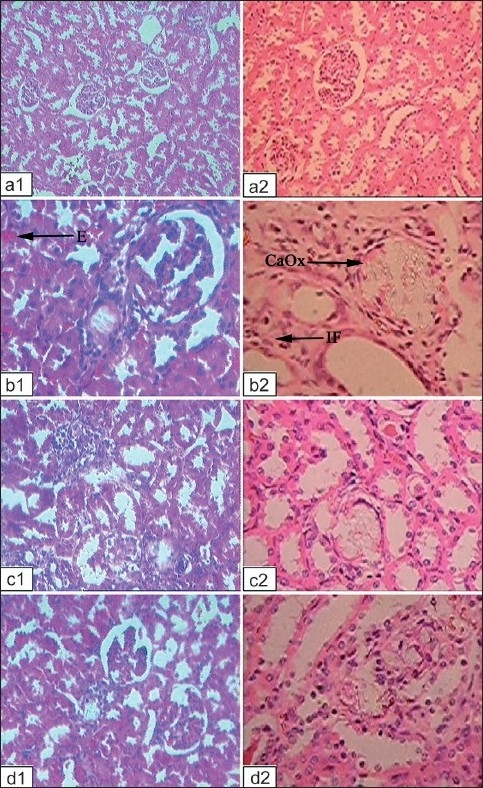
Microscopic images of kidney sections from (a) vehicle control animals, (b) lithiatic group, (c) group treated with rutin and (d) group treated with curcumin. 1 and 2 in the subscript reflect sections under polarized light microscope (100×) after Hematoxylin and Eosin staining and under light microscope after Pizzolato’s staining (40×), respectively. Lithiatic animals showed excessive accumulation of calcium oxalate crystals (CaO×) with marked histological changes including interstitial fibrosis (IF) with infiltration by eosinophils (E) (AT COLUMN WIDTH)

The kidney of calculi-induced rats showed marked histological changes including interstitial fibrosis with dense infiltration by eosinophils. Animals treated with rutin and curcumin showed minimal kidney damage [Figure 1] and histological changes compared to the kidneys of calculi-induced animals [Figure 2].

## DISCUSSION

Urinary supersaturation with respect to stone-forming constituents is generally considered to be one of the causative factors in calculogenesis. Renal calcium oxalate deposition by ethylene glycol and ammonium chloride in rats is frequently used to mimic the urinary stone formation in humans.[7] The biochemical mechanisms for this process are related to an increase in the urinary concentration of oxalate. Stone formation in ethylene glycol-fed animals is caused by hyperoxaluria, which causes increased renal retention and excretion of oxalate.[2] Ammonium chloride has been reported to accelerate lithiaisis.[16,17] Therefore, this model was used to evaluate the effect of rutin and curcumin on calcium oxalate urolithiasis.

Consistent with some previous reports, stone induction by EG and ammonium chloride caused an increase in calcium and oxalate excretion.[10] It is accepted that hyperoxaluria is a significant risk factor in the pathogenesis of renal stones. Increased urinary calcium is a factor favoring the nucleation and precipitation of calcium oxalate from urine and subsequent crystal growth.[2] Co-treatment with rutin and curcumin reduced the rate of increase in the calcium and oxalate excretion.

Increase in calcium and oxalate levels in the renal tissue of EG-treated rats was observed. The rutin and curcumin suppresses this increase in intracellular calcium. This might be due to the intracellular bioavailability of NO (nitric oxide) which in turns activates cGMP (3’,5’ cyclic guanosine monophosphate) that controls the increase in intracellular calcium levels. Previous studies reported that NO donors have the capacity to control the intracellular rise in calcium levels. Thus, rutin and curcumin could effectively control the levels of both calcium and oxalate by the following mechanism: (1) inhibiting the synthesis of oxalate and (2) increasing the bioavailability of NO to sequester calcium through the cGMP pathway.[10]

In urolithiasis, the glomerular filtration rate (GFR) decreases due to stones in the urinary system obstructing urine outflow, this leads to the accumulation of waste products in the blood, particularly nitrogenous substances such as urea, creatinine and uric acid. In addition, increased lipid peroxidation has been reported in the kidneys of calculi-induced rats. In this context, oxalate has been reported to induce lipid peroxidation and to cause renal tissue damage by reacting with polyunsaturated fatty acids in cell membranes.[10] In the present study, calculi-induced rats were found to have marked renal damage, consistent with the elevated serum level of creatinine. The administration of rutin and curcumin inhibited these changes that would otherwise promote new stone formation in the urinary system. The significant lowering of the serum creatinine level is attributed to an enhanced GFR and the anti-lipid peroxidative property of the rutin and curcumin.

Calcium oxalate crystals and high oxalate levels in nephrons can produce damages in the epithelial cells, and consequently, the cells may produce some products, as well as free radicals, inducing heterogeneous crystal nucleation and causing aggregation of crystals.[4] Several studies have reported that flavonoids, especially rutin and curcumin, have potent anti-inflammatory and antioxidant effects. Therefore, the role of rutin and curcumin in preventing formation of calcium oxalate urolithiasis, as seen in the present study, is in part due to their anti-inflammatory and antioxidant effects. These compounds may interfere with the process of epithelial cell damage induced by calcium oxalate crystals or may exert inhibitory effect on the inflammation.

In conclusion, the present study provides evidence that the oral administration of rutin and curcumin inhibits the development but failed to reverse the changes caused by calcium oxalate urolithiasis. Therefore, rutin and curcumin may be effective to prevent calcium oxalate urolithiasis than its treatment. In this study, we have not evaluated the effect of the combination of rutin and curcumin on calcium oxalate urolithiasis. Further research is necessary to investigate the mechanism underlying this inhibitory effect of rutin and curcumin and the effect of the combination of these two compounds on calcium oxalate urolithiasis.

## References

[1] Hadjzadeh MA, Khoei A, Hadjzadeh Z, Parizady M (2007). Ethanolic extract of Nigella Sativa L seeds on ethylene glycol-induced kidney calculi in rats. Urol J.

[2] Karadi RV, Gadge N, Alagawadi KR, Savadi RV (2006). Effect of Moringa oleifera Lam. root-wood on ethylene glycol induced urolithiasis in rats. J Ethnopharmacol.

[3] Liu J, Cao Z, Zhang Z, Zhou S, Ye Z (2007). A comparative study on several models of experimental renal calcium oxalate stones formation in rats. J Huazhong Univ Sci Technolog Med Sci.

[4] Thamilselvan S, Khan SR, Menon M (2003). Oxalate and calcium oxalate mediated free radical toxicity in renal epithelial cells: Effect of antioxidants. Urol Res.

[5] Selvam R (2002). Calcium oxalate stone disease: Role of lipid peroxidation and antioxidants. Urol Res.

[6] Itoh Y, Yasui T, Okada A, Tozawa K, Hayashi Y, Kohri K (2005). Preventive effects of green tea on renal stone formation and the role of oxidative stress in nephrolithiasis. J Urol.

[7] Bashir S, Gilani AH (2009). Antiurolithiatic effect Bergenia ligulata rhizome: An explanation of the underlying mechanisms. J Ethnopharmacol.

[8] Janbaz KH, Saeed SA, Gilani AH (2002). Protective effect of rutin on Paracetamol-and CCl4-induced hepatotoxicity in rodents. Fitoterapia.

[9] Maheshwari RK, Singh AK, Gaddipati J, Srimal RC (2006). Multiple biological activities of curcumin: A short review. Life Sci.

[10] Divakar K, Pawar AT, Chandrasekhar SB, Dighe SB, Divakar G (2010). Protective effect of the hydro-alcoholic extract of Rubia cordifolia roots against ethylene glycol induced urolithiasis in rats. Food Chem Toxicol.

[11] Kuhad A, Pilkhwal S, Sharma S, Tirkey N, Chopra K (2007). Effect of curcumin on inflammation and oxidative stress in cisplatin-induced experimental nephrotoxicity. J Agric Food Chem.

[12] Hodgkinson A (1970). Determination of oxalic acid in biological material. Clin Chem.

[13] Shah ZA, Gilani RA, Sharma P, Vohara SB (2005). Cerebroprotective effect of Korean ginseng tea against global and focal models of ischemia in rats. J Ethnopharmacol.

[14] Pizzolato P (1971). Mercurous nitrate as a histochemical reagent for calcium phosphate in bone and pathological calcification for calcium oxalate. Histochem J.

[15] Rothermund L, Luckert S, Koßmehl P, Paul M, Kreutz R (2001). Renal endothelin ETA/ETB receptor imbalance differentiates salt-sensitive from salt-resistant spontaneous hypertension. Hypertension.

[16] Fan J, Michael AG, Chandhoke PS (1999). Impact of ammonium chloride administration on a rat ethylene glycol urolithiaisis model. Scanning Microsc.

[17] Atmani F, Slimani Y, Mimouni M, Hacht B (2003). Prophylaxis of calcium oxalate stones by Herniaria hirsute on experimentally induced nephrolithiasis in rats. BJU Int.

